# Web-based health Information Seeking and eHealth Literacy among College students. A Self-report study

**DOI:** 10.17533/udea.iee.v38n1e08

**Published:** 2020-02-26

**Authors:** Fatemeh KHademian, Mahsa Roozrokh Arshadi Montazer, Azam Aslani

**Affiliations:** 1 Ph.D. Candidate. School of Management and Medical Informatics, Shiraz University of Medical Sciences, Shiraz, Iran. Email: fkhademian90yahoo.com Shiraz University of Medical Sciences Shiraz Iran fkhademian90yahoo.com; 2 M.Sc. Candidate. School of Management and Medical Informatics, Shiraz University of Medical Sciences, Shiraz, Iran. Email: mahsaroozrokh@yahoo.com Shiraz University of Medical Sciences Shiraz Iran mahsaroozrokh@yahoo.com; 3 Assistant professor, School of Management and Medical Informatics, Shiraz University of Medical Sciences, Shiraz, Iran. Email: aslaniaz@sums.ac.ir Shiraz University of Medical Sciences Shiraz Iran aslaniaz@sums.ac.ir

**Keywords:** consumer health information, telemedicine, students, health occupation, health literacy, Internet., información de salud al consumidor, telemedicina, estudiantes del área de la salud, alfabetización en salud, Internet., informação de saúde ao consumidor, telemedicina, estudantes de ciências da saúde, alfabetização em saúde, Internet.

## Abstract

**Objective.:**

This study aimed to assess web-based health information seeking and eHealth literacy among Iranian college students.

**Methods.:**

The study was conducted in five colleges of the Shiraz University of Medical Sciences in Iran during 2018. The data were collected by a researcher-made questionnaire consisting of seven questions on a 4-point Likert-type scale, with scores ranging from 7 to 28. These questions were: ′I know how to use the Internet to answer my questions about health′, ′I think there is enough information about health-related issues on the Internet′, ′I know the vocabulary used in health issues on the Internet′, ′I can tell high-quality health resources from low-quality health resources on the Internet′, ′I know how to use the health information I find on the Internet to help me′, ′I feel confident in using information from the Internet to make health decisions′, and ′Searching for health-related information on the Internet will increase my knowledge in this field′. High eHealth literacy level is defined as above the total mean score and low eHealth literacy level is defined as lower than the total mean score.

**Results.:**

In all, 386 college students participated in the study. The results showed that the mean score of eHealth literacy was 19.11 out of 28; 205 participants (54.4%) had low eHealth literacy. In addition, the students used the Internet to search for information regarding diseases symptoms (70%), physical illnesses (67.1%), existing treatments (65%), and diagnosis (63.1%).

**Conclusion.:**

The results showed that participants in this study usually searched for illnesses, symptoms, and treatments after they got sick and paid little attention to other aspects related to integral health.

## Resumen

**Objetivo.:**

Evaluar la búsqueda de información de salud en línea y el nivel de alfabetización en eSalud entre los estudiantes universitarios iraníes.

**Métodos.:**

El estudio se realizó en cinco colegios de la Universidad de Ciencias Médicas de Shiraz, Irán, durante 2018. Los datos se recopilaron con la ayuda de un cuestionario realizado por los investigadores que consta de 7 afirmaciones con opciones de respuesta tipo Likert de 4 puntos, con un rango de puntaje de 7-28 puntos: 1. “Sé cómo usar Internet para responder mis preguntas sobre salud”; 2. “Creo que hay suficiente información sobre problemas relacionados con la salud en Internet”; 3. “Conozco el vocabulario utilizado en temas de salud en Internet”; 4. “Puedo distinguir en Internet los recursos de salud de alta calidad de los que son de baja calidad”; 5. “Sé cómo usar la información de salud que encuentro en Internet para ayudarme”; 6. “Me siento seguro al usar la información de Internet para decisiones de salud”, y 7. “Buscar en Internet información relacionada con la salud aumentará mi conocimiento en este campo”. Se estableció que se tenía alfabetización en eSalud alta si el puntaje estaba por encima de la media total y alfabetización en eSalud baja si este puntaje era inferior a la puntuación media.

**Resultados.:**

386 estudiantes universitarios participaron en el estudio. La puntuación media de alfabetización en eSalud fue de 19,11 de los 28 puntos máximos posibles. 205 participantes (54.4%) tenían baja alfabetización en eSalud. Además, los estudiantes utilizaron Internet para buscar información sobre síntomas de enfermedades (70%), enfermedades físicas (67.1%), tratamientos existentes (65%) y diagnóstico (63.1%).

**Conclusión.:**

Los resultados mostraron que los participantes de este estudio buscaban generalmente información en Internet acerca de enfermedades, síntomas y tratamientos después de enfermarse y prestaban poca atención a otros aspectos relacionados con la salud integral.

## Resumo

**Objetivo.:**

Este estudo teve como objetivo avaliar a busca de informações sobre saúde on-line e o nível de alfabetização em eSaúde entre estudantes universitários iranianos.

**Métodos.:**

O estudo foi realizado em cinco faculdades da Universidade de Ciências Médicas de Shiraz, Irã, durante 2018. Os dados foram coletados com a ajuda de um questionário conduzido pelos pesquisadores, composto por 7 declarações com opções de resposta tipo Likert de 4 pontos, com uma faixa de pontuação de 7-28 pontos. Essas perguntas foram: 'Eu sei como usar a Internet para responder às minhas perguntas sobre saúde', 'Eu acho que há informações suficientes sobre problemas relacionados à saúde na Internet', 'Eu conheço o vocabulário usado nas questões de saúde na Internet', 'Eu posso distinguir em Recursos de saúde de alta qualidade na Internet que são de baixa qualidade ',' Eu sei como usar as informações de saúde encontradas na Internet para me ajudar ',' Sinto-me seguro ao usar informações da Internet para decisões de saúde 'e' A busca de informações relacionadas à saúde na Internet aumentará meu conhecimento neste campo '. Foi estabelecido que havia alta alfabetização em eSaúde se a pontuação estivesse acima da média total e baixa literacia em eSaúde se essa pontuação fosse menor que a média.

**Resultados.:**

386 universitários participaram do estudo. A pontuação média em alfabetização em eSaúde foi 19,11 dos 28 pontos máximos possíveis. 205 participantes (54,4%) tinham baixa alfabetização em eSaúde. Além disso, os estudantes usaram a Internet para buscar informações sobre sintomas da doença (70%), doenças físicas (67,1%), tratamentos existentes (65%) e diagnóstico (63,1%).

**Conclusão.:**

Os resultados mostraram que os participantes deste estudo geralmente buscavam informações na Internet sobre doenças, sintomas e tratamentos após adoecer e prestavam pouca atenção a outros aspectos relacionados à saúde integral.

## Introduction

Internet is being used increasingly in the world and almost half of the world’s populations are Internet users. The rate of Internet usage increased by 933.8% in the world from 2000 to 2017.(1) The increasing use of the Internet and mobile technology has made it possible to have access to information at any time and any place.(2) Indeed, people can stay in contact with each other anywhere in the world. In 2016, 88% of adults in the United States used the Internet, 99% of whom were between 18 and 29 years old.(3) Iran is ranked 13th in the world in terms of number of Internet users and about 70% of Iranians use the Internet.(1) People can have access to data and information via Internet in a secure, easy, cheap, and fast way.(4) Moreover, Internet is considered a main source for finding health information.(5)

In addition to having access to the Internet, special skills are needed for using and evaluating electronic recourses(6) because quality, reliability, and accuracy of health information resources are poor in some cases.(7,8) In other words, people may access inaccurate health information that is potentially dangerous if they do not have adequate skills. Thus, people should have enough skills to evaluate the information on the Internet. In fact, people should have a good level of eHealth literacy. eHealth literacy refers to “the ability of individuals to seek, find, understand, and appraise health information from electronic resources and apply such knowledge to addressing or solving a health problem”.(9) The results of a study showed that many college students did not have enough eHealth literacy skills.(10) Poor usability of eHealth services can cause barriers against access to and use of on-line health information. Therefore, eHealth literacy skill tools have to be improved.(11) eHealth literacy results in good health behaviors and positive changes.(12,13)

The results of a review study indicated that the students did not have enough Internet literacy skills and had to acquire the necessary skills.(10) A similar study was conducted to assess on-line health literacy of nursing students in South Korea. In that study, most participants reported that the Internet was a useful source to make health-related decisions, but only a small number of them were able to distinguish between high-quality and low-quality sources.(5) Another study showed that 71% of the participants were eager to use the Internet to search for health-related issues. Meanwhile, more than half of the participants (52%) had a good Internet health literacy level. That study also revealed the importance of knowing the credible sources of information. Accordingly, the participants who had heard the name of MedlinePlus database had higher levels of Internet health literacy.(14) The results of another study showed that despite being aware of the Internet resources and searching the Internet, students had problems in evaluating these sources and distinguishing between high-quality and low-quality sources. In that study, the factors related to Internet health literacy included the type of university, type of student admission, level of education, students’ online skills, and their understanding of the importance and usefulness of the Internet. However, there was no significant association among eHealth literacy and age, gender, and frequency of Internet usage.(15) The results from another study showed that 53% of students tended to seek for health information on the Internet and 74% sought for health information on the Internet. Additionally, the most important challenge for the students was the accuracy of on-line information on the Internet.(16) Other studies demonstrated that students’ average of actual Internet health literacy was significantly lower than their average perceived Internet literacy. In addition, students with higher education levels had higher levels of Internet health literacy compared to those with lower levels of education.(17)

Given that most people obtain the necessary health-related information from the Internet and it is difficult and even impossible to check all the information available on websites and weblogs, users themselves should have the ability to check the quality of the information. The present study sought to investigate eHealth literacy level among college students in Shiraz, Iran.

## Methods

This cross-sectional, descriptive study was conducted in five colleges of the Shiraz University of Medical Sciences, Shiraz, Iran from April to May 2018. The inclusion criteria of the study were having an associate degree, BSc/MSc student, studying in one of the selected colleges at the time of data collection, and being willing to participate in the study. The individuals who did not fill out the questionnaire completely were excluded from the study. 

The data were collected by a questionnaire devised by researchers based on the literature review. The face and content validity of the questionnaire was confirmed by the experts and its reliability was confirmed by a Cronbach’s alpha of 0.78. It was a self-report tool that assessed the students’ perceptions of their skills and knowledge within each measured domain. The main part of the questionnaire, i.e., eHealth literacy domain consisted of seven questions that were answered on a four-point Likert scale, from strongly disagree (score = 1) to strongly agree (score = 4). The total scores ranged from 7 to 28. Furthermore, the questionnaire included six supplementary parts including demographic characteristics, frequency of using the Internet for different items, use of electronic and non-electronic sources, factors affecting the evaluation of health websites, reasons for using the Internet, and evaluating the experience of using the Internet. 

The researcher provided the list of five colleges of the university, including nursing, management and medical informatics, health and nutrition, rehabilitation, and paramedical schools. Considering the content of courses and the possibility of higher eHealth literacy level of dentistry, medicine, and pharmacy students compared to other students, they were excluded and those from other five colleges were invited to participate in the study. The number of participants from each college was determined based on its total number of students. After obtaining approval from the university administration, the researcher visited each college on random days and invited the students who were available and gave them a brief explanation about the study objectives and procedures. After that, the students who were willing to participate in the study signed the informed consent and filled out the questionnaires. Overall, 402 students completed the questionnaires. 

The study data were analyzed using the Statistical Package for the Social Sciences (SPSS), version 16. Descriptive statistics was used to describe variables. Spearman’s correlation test was used to assess the relationship between age and eHealth literacy. Additionally, to compare eHealth literacy mean scores based on type of college, education level (i.e., associate degree, BSc, MSc), living condition (i.e., alone, with family, in dormitory), and frequency of computer and laptop use per week (i.e., 1-2 hours, 3-4 hours, 5-6 hours, more than 6 hours), Kruskal-Wallis test was used. To compare eHealth literacy scores based on sex, marital status, and type of residence, the Mann-Whitney test was used.

Ethics approval and consent to participate. This study was approved by the Ethics Committee of the Shiraz University of Medical Sciences (code: 1397.067). Data collection was started after obtaining a formal authorization from the University’s Ethics Committee. Participation in this study was entirely voluntary. Besides, the students were reassured about the confidentiality of their information. In other words, the participants were identified by using a unique identification code.

## Results

A total of 402 students completed the questionnaires, but those with more than 10% missing items were put aside. After all, 16 questionnaires were excluded and 386 cases were analyzed [School of Nursing and Midwifery (31.4%, *n*=121), School of Management and Medical Informatics (22.3%, n=86), School of Health and Nutrition (18.7%, *n*=72), School of Rehabilitation (16.9%, *n*=65), and Paramedical School (10.6%, *n*=41)]. The mean age of the participants was 22.25 (SD= 2.48) years. In addition, most of the participants were female (94%), BSc students (89.3%), single (83.9%), and urban residents (86.8%). Besides, most of the students lived in dormitories (69.1%). Moreover, nearly half of the students (51.8%) used the computer for 1-2 hours daily. Moreover, the students mostly used the Internet in dormitories (81.3%), home (39%), university (15.5%), and coffee shops and public places (3.6%). The first priority of the students about the question “Who requires the health-related information?” was as follows: myself (82.1%), family (75.6%), and friends and colleagues (30.3%). 

The result showed that the mean score of eHealth literacy was 19.11(SD=2.96) out of 28. Based on the previous studies (3.17) the level of eHealth literacy was measured according to the total mean score. Thus, scores above the total mean score (i.e., >19) were considered high eHealth literacy level and scores equal to mean score and lower than it (i.e., ≤19) were considered low eHealth literacy level. Accordingly, most participants’ eHealth literacy level was low (54.4%, *n*=205). The students’ eHealth literacy scores have been presented in Table 1. As the table depicts, the participants gained low scores in recognizing high-quality from poor-quality information and trusting the information found on the Internet and using it to make decisions. 

**Table 1:** eHealth literacy among the students

eHealth statements	Strongly disagree n (%)	Disagree n (%)	Agree n (%)	Strongly agree n (%)	Item Mean (SD)
I know how to use the Internet to answer my questions about health.	8 (2.1)	60 (15.5)	251 (65.0)	67 (17.4)	2.98 (0.64)
I think there is enough information about health-related issues on the Internet.	14 (3.6)	90 (23.4)	243 (63.1)	38 (9.9)	2.79 (0.66)
I know the vocabulary used in health issues on the Internet.	15 (3.9)	151 (39.2)	200 (51.9)	19 (4.9)	2.58 (0.64)
I can tell high-quality health resources from low-quality health resources on the Internet.	21 (5.5)	148 (38.3)	186 (48.3)	30 (7.8)	2.58 (0.71)
I know how to use the health information I find on the Internet to help me.	10 (2.6)	90 (23.4)	262 (68.1)	23 (6)	2.77 (0.58)
I feel confident in using information from the Internet to make health decisions.	26 (6.8)	171 (44.8)	172 (45)	13 (3.4)	2.45 (0.67)
Searching for health-related information on the Internet will increase my knowledge in this field.	15 (3.9)	30 (7.8)	290 (75.1)	50 (13)	2.97 (0.60)

The students’ answers to the question “How many times have you used the Internet for the following items over the past six months?” are shown in Table 2. Accordingly, 70% of the students stated that they used the Internet to search for information about disease symptoms (every day, 1-2 times a month). Additionally, 79.2% of the participants reported that they did not use (never, rarely) the Internet to contact physicians or other healthcare providers.

**Table 2:** The rate of internet use among the students over the past six months

Questions	Every day n (%)	1 time per week n (%)	1 to 2 times a month n (%)	Rarely n (%)	Never n (%)
Information related to physical illness	37 (9.6)	109 (28.2)	113 (29.3)	108 (28)	19 (4.9)
Information on mental health, depression, and stress	13 (3.4)	54 (14)	98 (25.4)	149 (38.6)	72 (18.7)
Prevention	21 (5.5)	72 (18.8)	106 (27.7)	135 (35.3)	48 (12.6)
Diagnosis	24 (6.3)	79 (20.7)	135 (35.3)	111 (29.1)	33 (8.6)
More information on the symptoms of the disease	35 (9.3)	80 (21.2)	149 (39.5)	89 (23.6)	24 (6.4)
Information on the results of medical tests	20 (5.3)	52 (13.8)	103 (27.2)	150 (39.7)	53 (14)
Information on existing treatments	27 (7)	78 (20.3)	145 (37.7)	111 (28.8)	24 (6.2)
Side effects of medications or treatments	26 (6.8)	64 (16.8)	146 (38.3)	109 (28.6)	36 (9.4)
Operational care information (bathing, first aid, etc.)	18 (4.8)	41 (10.9)	105 (27.9)	151 (40.2)	61 (16.2)
Information about lifestyle (nutrition, exercise, dietary habits, physical activity, smoking, alcohol consumption, etc.)	32 (8.3)	73 (19)	122 (31.8)	116 (30.2)	41 (10.7)
Information about caring for an elderly person	6 (1.6)	17 (4.5)	41 (10.8)	142 (37.4)	174 (45.8)
News related to health policies, such as insurance costs, medications, visits, and more	11 (2.9)	25 (6.6)	51 (13.5)	144 (38)	148 (39.1)
Information related to a physician, hospital, nursing home, home care center, or other care providers	10 (2.6)	24 (6.3)	71 (18.5)	149 (38.8)	130 (33.9)
How to adapt to the disease	9 (2.3)	25 (6.5)	92 (24)	150 (39.1)	108 (281.)
Emotional support in dealing with a health issue	14 (3.7)	26 (6.9)	87 (23)	132 (34.8)	120 (31.7)
To collaborate with other patients through social networks	8 (2.1)	21 (5.5)	53 (13.8)	134 (35)	167 (43.6)
To contact a physician or other healthcare providers, by E-mail, etc.)	11 (2.9)	17 (4.5)	51 (13.4)	119 (31.2)	183 (48)
For general studying of health or diseases without a specific purpose	22 (5.7)	48 (12.4)	106 (27.5)	124 (32.1)	86 (22.3)

The results related to responses to the question “In which sources do you find health-related information?” are presented, thus: 70.9%, search engines like Google and Yahoo; 26.3%, mobile apps; 24.2%, on-line social networks; 18.3%, blogs and related specialized websites; 16.4, websites from official health organizations, such as the Ministry of Health and WHO; and 9.6%, Internet magazines and newspapers.

In order to determine whether the students made necessary assessments when using Internet resources, some questions were asked, as shown in Table 3. Accordingly, updated information, being approved by a physician, protection of users’ personal information, and presence of healthcare professionals were among the most important factors. However, being recommended by a friend or family member was not very important for most students. 

**Table 3:** The participants’ perspective on the criteria for evaluating health websites

Questions	Not important n (%)	A little important n (%)	Somewhat important n (%)	Important n (%)	Very important n (%)
Protecting users’ personal information	17 (4.4)	24 (6.2)	46 (11.9)	117 (30.3)	182 (47.2)
Information in my language	9 (2.3)	18 (4.7)	56 (14.5)	157 (40.7)	146 (37.8)
Updated information	3 (0.8)	11 (2.9)	24 (6.3)	86 (22.5)	259 (67.6)
Interaction (such as answering / chat / chat services)	17 (4.5)	28 (7.4)	99 (26.1)	123 (32.4)	113 (29.7)
The presence of healthcare professionals	9 (2.4)	24 (6.3)	48 (12.7)	116 (30.6)	182 (28)
Clarity of site officials and sponsors	49 (12.8)	47 (12.3)	106 (27.7)	81 (21.2)	99 (25.9)
The proper design of the site	13 (4.7)	61 (15.9)	113 (29.5)	109 (28.5)	82 (21.4)
Link to other sites	28 (7.3)	59 (15.4)	116 (30.2)	114 (29.7)	67 (17.4)
Specialized resources	7 (1.8)	19 (5)	57 (14.9)	144 (37.6)	156 (49.7)
Approved by a physician	11 (2.9)	23 (6)	56 (14.5)	104 (27)	191 (49.6)
Recommended by a friend or family member	49 (12.8)	95 (24.7)	131 (34.1)	66 (17.2)	43 (11.2)

In this study, the relationship between eHealth literacy scores and different variables was examined. The findings of the Spearman correlation test did not show any statistically significant correlation between eHealth literacy score and age (*r=*0.07, *p*=0.17). Furthermore, the results of the Mann-Whitney test showed no statistically significant difference between the mean score of eHealth literacy and sex (*p*=0.17), marital status (*p*=0.66), and type of residence (*p*=0.08). Additionally, the result of the Kruskal-Wallis test showed no statistically significant difference between eHealth literacy scores and type of college (*p*=0.30), education level (*p*=0.39), living condition (*p*=0.13), and frequency of computer and laptop use per week (*p*=0.32). 

The next part of the questionnaire was about the “reasons for using the Internet from the students’ point of view”, and the results state that the majority of the participants (95.8%) either agreed or strongly agreed with easy access to information. Also, more than half of the participants (90.7%) agreed and strongly agreed with access to extensive information from various sources. Other reasons included quick access to information (94.3%), the possibility of studying various and shameful topics privately (69.5%), cheaper Internet information than the cost of physician’s visit (72.5%), advice from a physician or other healthcare providers, such as nurses, midwives, and radiologists (43.3%), and being recommended by a friend or family member (43.5%). 

Most of the students were opposed to the high cost of searching the Internet (77.4%), inaccessibility of information in their language (65.5%), and inability to find what they looked for (75.6%). On the other hand, they believed that the information they searched for was helpful (91.6%) and easy to understand (90.1%). The majority of the participants (90.1%) reported that they were much or very much likely to use the Internet the next time they needed health information. Also, 95.8% of the students stated that the Internet was a good tool to improve knowledge about health-related issues. Furthermore, 89.1% of the participants reported that they were generally satisfied with the information they found on the Internet.

## Discussion

The study results showed that the mean score of eHealth literacy was 19.06 out of 28. Indeed, the students used the Internet as a good source to search for health-related information. Based on the results, most of the participants used the Internet to search for information on disease symptoms, physical illnesses, existing treatments, and diagnosis, respectively. In Most cases, they believed that they knew how to use the health information they found on the Internet. These results are supported by another study on young Italians, which indicated that 60% of males and 65% of females used the Internet for health-related purposes.(18) In the present study, the participants were asked about their perspective on evaluating health sites on the Internet. These questions were then matched with Health Summit Working Group’s (HSWG) criteria. The results demonstrated that some criteria, including protecting users’ personal information, existence of information in their language, updated information, presence of healthcare professionals, and being approved by a physician, were important for the students.

In this study, most participants’ eHealth literacy level was low (54.4%). Similar to this finding, the health literacy of Iranian medical and other health sciences university students was low.(19) However, Tubaishat *et al*.,(15) reported that 51.1% of university students in South Korea had high eHealth literacy. The different findings may be related to the difference between target populations of these two studies. In the present study, the participants were from different fields in a public university, whereas in the study by Tubaishat *et al*.(15) only nursing students in public and private universities had participated. Another study using effective educational strategy recommended improving nursing students’ learning outcomes and self-efficacy or belief on their ability to succeed in achieving learning goals.(20) Therefore, applying effective strategies to improve health-related university students’ eHealth literacy is suggested. 

In the current study, 47.9% of the participants did not search the net for prevention-related information. Besides, most of the participants rarely or never searched the Internet for information on mental health, depression, and stress, emotional support in dealing with a health issue, taking care of an elderly person and news related to health policies, such as insurance costs, medications, and visits. These results reflect a disease-centered perspective vs. health-centered perspective among Iranian health-related university students. In addition, their searches were mostly focused on physical illnesses and other aspects of health, especially mental health, is underestimated. In addition, they paid little attention to community-based aspects of health. These findings indicate that they need to learn about the more comprehensive perspective of health and healthcare issues in which all aspects of health, particularly mental health, are emphasized. Additionally, they need to learn about the importance of community-based issues of healthcare, such as prevention, policies, costs etc. In this regard, mobile-based applications, such as mental health apps, may be used to improve their knowledge and attitudes about these important issues.(21) 

The participants also rarely or never searched for ways to adapt to diseases, collaboration with other patients through social networks, and contact with a physician or other healthcare providers. These results agree with those in the study by Bhandari *et al.*(22) in the United States. They found that a small proportion of the participants (4%) communicated with physicians through e-mail. These findings indicate that there are barriers in applying some kinds of telemedicine, such as communicating with physicians and other healthcare providers through e-mail. These barriers may be different from subjective obstacles to the lack of technical infrastructures.

In the current study, most of the respondents reported using search engines, like Google and Yahoo, for surfing the net, which is consistent with another study.(23) Findings showed that about half of the students could not distinguish high-quality from low-quality health resources on the Internet, and did not feel confident in using the information obtained from the Internet to make health decisions. Similarly, Rathnayake *et al.*(10) found that only 17.03% of their respondents were confident to use on-line health information to make health decisions and 41.1% of them had the ability to distinguish between high- and low-quality health resources on the Internet. These measurements were, respectively, obtained as 28.6% and 21.6% in the research by Tenant *et al*.(24)

Although the current study’s participants used the Internet as a good source in most participants, it did not lead to decision-making in about half of them. It seems that they sought information for educational purposes rather than for decision making. Moreover, 43.1% of the on-line resources that students searched were not understandable to them and they could not understand the vocabulary used in these resources. Thus, readily available and validated tools are recommended to be designed to assess the readability of written materials to create understandable materials for the target population.

Strengths and limitations. This study had strengths, such as the large sample size and that of conducting the study on students from five different colleges. On the other hand, it had some limitations. Firstly, given that simple random sampling could not be used, the days of the week were randomized. Secondly, the study data were collected by using questionnaire and self-report data could be affected by the students’ subjectivity.

## Conclusion.

The study results showed the pattern of search among Iranian students who usually searched for illnesses, symptoms, and treatments after they got sick. Thus, health policymakers are required to design patient-centered health websites in a language that is understandable to the community. Additionally, the content must be written in a way that helps people make decisions about their illnesses. It is also necessary to plan seriously for increasing the eHealth literacy level of the community, especially students. In addition, it is essential to clearly explain the indicators of trusting the Internet content to users. This study revealed the similarities of health literacy among different countries regardless of their development level, which implies the generalizability of the results.

Overall, the study findings provided useful information for decision-makers to provide more efficient educational programs. Given the opportunities that the Internet has provided for better education, it is suggested that students’ Internet literacy skills should be improved. In addition, eHealth literacy skills are recommended to be embedded into the students’ curricula. 
